# Electric Field-Defined Superlattices in Bilayer Graphene: Formation of Topological Bands in Two Dimensions

**DOI:** 10.3390/ma18071521

**Published:** 2025-03-28

**Authors:** Włodzimierz Jaskólski

**Affiliations:** Institute of Physics, Faculty of Physics, Astronomy and Informatics, Nicolaus Copernicus University in Toruń, Grudziądzka 5, 87-100 Toruń, Poland; wj@fizyka.umk.pl

**Keywords:** multilayer graphene, topological states, superlattices

## Abstract

An electric field applied to the Bernal-stacked bilayer graphene opens an energy gap; its reversal in some regions creates domain walls and leads to the appearance of one-dimensional chiral gapless states localized at the walls. Here, we investigate the energy structure of bilayer graphene with superlattice potential defined by an external electric field. The calculations are performed within an atomistic π-electron tight-binding approximation. We study one-dimensional and two-dimensional superlattices formed by arrays of electric-field walls in the zigzag and armchair directions and investigate different field polarizations. Chiral gapless states discretize due to the superlattice potential and transform into minibands in the energy gap. As the main result, we show that the minibands can cross at the Fermi level for some field polarizations. This leads to a new kind of two-dimensional gapless states of topological character that form Dirac-like cones at the crossing points. This also has application potential: changing the field polarization can close the energy gap and change the character of the superlattice from semiconducting to metallic.

## 1. Introduction and Model Description

Gated Bernal-stacked bilayer graphene (BLG) has attracted attention over the past twenty years as a platform for studying one-dimensional chiral gapless states of topological character. These states usually appear at stacking boundaries [1,2,3,4,5,6,7,8,9,10,11] but also in stacking change-free multilayers at interfaces separating regions of different gate polarizations [8,12,13,14,15,16]. This way of creating domain walls with one-dimensional gapless states has an advantage over the formation due to the stacking change. It does not cause problems with strain and is not limited to specific directions. Changes in stacking play also an important role in twisted multilayers, where flat bands at the Fermi level, strongly correlated phases and superconducting properties have been reported [17,18,19,20,21,22,23,24,25,26,27]. Recently, the idea of replacing twisted multilayers by systems with spatially modulated gates for studying topological flat bands has been proposed [28,29].

A simple reversal of the electric field across an interface line, i.e., an electric-field wall (EFW) separating two bilayer regions, creates at each valley a pair of topological one-dimensional and conducting bands that connect the valence and conduction-band continua [12,14]. One can apply more than one interface line with the reversed-field polarization and check how this influences the topological bands. Periodic arrays, i.e., superlattices (SL) of electric-field walls, lead to the creation of topological minibands in two dimensions [28,30,31,32,33,34]. The study of such minibands is the main aim of this paper.

**Figure 1:**
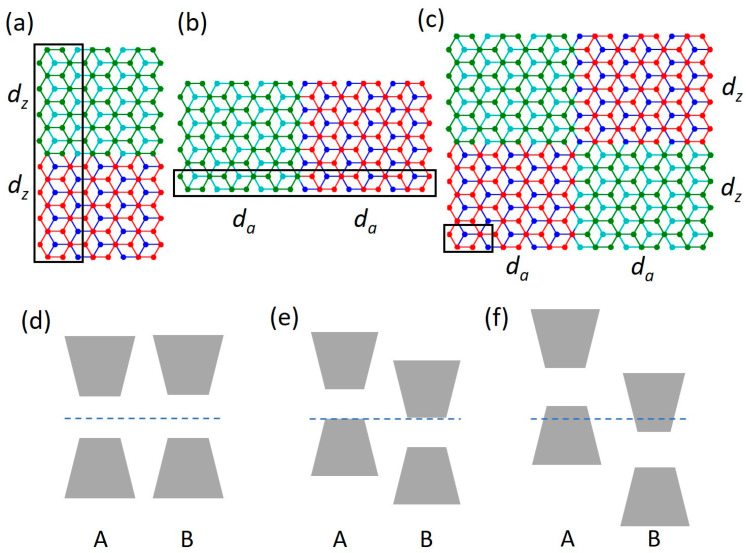
(**a**,**b**) Schematic representations of the 1D ASL and ZSL superlattices, respectively. Black rectangles mark the SL unit cells. (**c**) An example of a unit cell of the 2D SL. Different colors of BLG layers (green/cyan and red/blue) represent the A and B sections of the SL with different pairs of gates applied to the upper (U)/lower (L) layers. The size of the A/B sections is *d*_a/z_. All systems extend periodically in both directions. The black rectangle in (**c**) shows an 8-atom unit cell of the BLG. (**d**–**f**) schematic representations of three gate regimes R1, R2, and R3, showing the A and B sections’ energy spectra, if considered as separate BLGs. The potentials *V* applied to the lower (L) and the upper (U) layers of BLG are (**d**) VAL = −*V*, VAU = *V*, VBL = *V*, VBU = −*V*; (**e**) VAL = 0, VAU = 2*V*, VBL = 0, VBU = −2*V*; and (**f**) VAL = Δ, VAU = 2*V* + Δ, VBL = −Δ, VBU = −2*V* − Δ. The horizontal broken line at *E* = 0 represents the Fermi level.

We investigate band structures of one-dimensional and two-dimensional arrays of domain walls in bilayer graphene, created by a spatially modulated electric field. The perpendicular electric field results in different gates applied to the layers. The EFWs are arranged along the zigzag or/and armchair directions. The systems under study are schematically presented in Figure 1. Panels (a) and (b) show fragments of one-dimensional superlattices with EFW along the armchair (ASL) and zigzag (ZSL) directions, respectively. Panel (c) shows an example of a 2D SL unit cell. Different colors of BLG sublattices represent regions of the bilayer with different pairs of gates applied to the lower and upper layers (We apply a sharp gate voltage change at the interface. This is an approximation that is not exactly possible in an experiment. We make this simplification because we are interested in the structure of the SL minibands and not in the effects of the electric field shape at the interfaces. Nevertheless, further studies are needed to determine the influence of these effects, as well as the role of the SL geometry). The walls are separated by *d*, measured in eight-atom BLG cell units, so the 2D SL unit cell’s size equals 2*d*.

We go beyond a simple reversal of the gate polarization in the neighbor sections of the SL and investigate three different gate regimes. In the first one (R1), represented by Figure 1d, the gate voltages ±*V* applied to the lower and upper BLG layers are simply reversed in the neighbor sections of the SL; the corresponding energy gaps *E_g_* are also reversed. In the second regime (R2) (Figure 1e), an additional gate voltage V_0_ = *V* is applied to both layers in one SL section, and V_0_ = −*V* to both layers of the neighbor section. As a result, the conduction-band edge of one section fits the valence-band edge of the neighbor section. In the third regime (R3), shown in Figure 1f, another additional voltage ±Δ is applied to the neighbor sections, so the valence band of one section partially overlaps (by 2Δ) the conduction band of the other.

In the case of 1D SL, the metallic character along the EFWs is governed by chiral 1D gapless bands. In 2D SL, these bands disintegrate and transform into SL minibands. When the first gate regime is applied, the minibands are located in the main energy gap *Eg,* but the mini-gap around the Fermi level (*E_F_*) is sustained and the system is no longer metallic. In the other gate regimes, the SL minibands can stick together or even cross at *E_F_* away from the high-symmetry points in the mini BZ (mBZ). Semi-metallicity is recovered in two dimensions. The crossing points resemble the Dirac cones of graphene, but here they are of topological origin because they come from the chiral, gapless states of gated BLG.

## 2. Computational Methods

To investigate the electronic band structure of 1D and 2D SL, we perform calculations in the atomistic model, i.e., we use the π-electron tight-binding (TB) approximation [35]. The TB Hamiltonian is given by

$$H=t_{i/e}\sum_{<i,j>}c_{i}^{†}c_{j}+H.c.$$

where $c_{i}^{†}$, ($c_{i}$) are the creation and (annihilation) operators for electrons at site *i*. The index *i* goes over all the nodes in the SL unit cell and the summation <*i*,*j*> is restricted to nearest neighbors. Intra-layer and inter-layer standard TB hopping parameters $t_{i}=2.7$ eV and $t_{e}=0.27$ eV are used, respectively [36,37]. Periodic boundary conditions are provided by connecting nodes from opposite edges of the SL unit cell with $\vec{k}$-dependent $t_{i}$. The energy bands $E(\vec{k})$ are found by diagonalizing the TB Hamiltonian for $\vec{k}$ values along the selected paths in the mBZ. In the largest system considered, the 2D SL unit cell contains ≈9200 nodes, which is the dimension of the Hamiltonian matrix.

It is worth emphasizing that the TB approach allows us to go beyond the continuous models, in which the BLG Hamiltonians represent mainly the energies close to the valleys. Here, the contribution to the SL minibands comes from the entire BLG energy spectrum. On the other hand, the TB model allows for the description of systems whose unit cells contain thousands of atoms and therefore appears to be simpler than the DFT approaches widely used in the modeling of various 2D systems (see, e.g., [38]).

For the case of a single EFW, we also calculate the local density of states (LDOS) at the interface. We use the Green function-matching technique for systems built of a central section connected to two semi-infinite leads [39]. In our case, the central section consists of the EFW region and the leads are the BLG half-planes. The LDOS is calculated as

$$LDOS\left(E\right)=-\frac{1}{\pi }Im\left[TrG_{C}(E)\right]$$

where $G_{C}\left(E\right)={\left(E-H_{C}-S_{L}-S_{R}\right)}^{-1}$ is the Green function of the central section, $H_{C}$ is the corresponding TB Hamiltonian and $S_{L/R}$ are self-energies of the leads. Since the system is periodic along the EFW, it is sufficient to consider a single BLG unit cell in this direction. The local density of states is then *k*-dependent, i.e., LDOS(*E*,*k*), where *k* is the wave vector corresponding to this periodicity.

## 3. Results and Discussion

### 3.1. Single EFW

**Figure 2:**
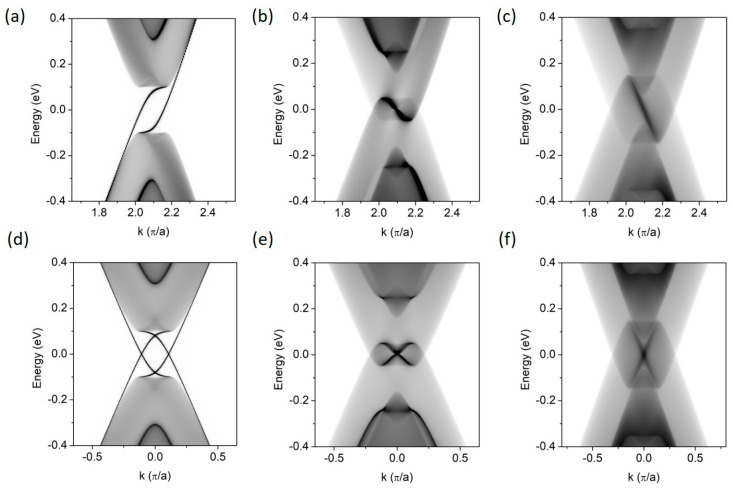
The energy spectra (LDOS) of a single EFW. (**a**–**c**) EFW in the zigzag direction; (**d**–**f**) EFW in the armchair direction. (**a**,**d**) correspond to R1, (**b**,**e**) to R2, and (**c**,**f**) to R3 gate regimes, respectively. *V* = 0.15 eV; Δ = 0.05 eV.

We first analyze the LDOS of a single EFW formed in BLG in the zigzag or armchair directions, for three gate regimes. The results are presented in Figure 2. The BLG is infinite in both directions but is periodic in the wall direction only. Figure 2a,d show the well-known energy structure of a single EFW with the gate voltage reversed across the zigzag and armchair directions, respectively [8,12]. In case (a) there is a mirror-symmetrical LDOS at −*K* valley, where *K* = π/a and *a* is the lattice constant along the EFW. In case (b), the valleys overlap at the Γ point and the gapless states with different slopes cross, since they are valley-protected.

In the second gate regime, Figure 2b,e, the absolute energy gap disappears, but some resonant states appear around the *E_F_*. For EFW along the zigzag direction, the resonance has a horizontal S-shape and is mirror-reflected at −*K*, while for the armchair direction, there are two overlapping S-shape resonances. In the third gate regime, Figure 2c,f, the resonance bands form straight lines with the same slopes as the BLG Dirac cones.

### 3.2. One-Dimensional Superlattices

To begin, let us take the first step and consider a system built of only one pair of parallel EFWs. The sequence of gate polarization reverses at each wall, so the mirror-reflected gapless states (*K* and −*K*) overlap in each valley. For very large wall separation, the gapless bands for both the ZSL and ASL cases look like they do in Figure 2d, but in the ASL case, they are doubly degenerate. The smaller wall spacing lifts some degeneracies at the band-crossing points, but the intrinsic band degeneracy in the ASL case is sustained.

We have calculated the energy structure of 1D SL with the wall spacing of *d*_a_ = 30 in the ASL case and *d*_z_ = 25 in the ZSL case. The value of *d*_a/z_ is measured in the units of the lengths u_a/z_ of an eight-atom graphene cell (see Figure 1c). Note, that u_a_ and u_z_ are different since u_a_ = a_C-C_ and u_z_ = 3a_C-C_. The lengths of the resulting ZSL and ASL unit cells are 220 Å and 153 Å, respectively. The BZ is a rectangle (2π/*d*_a/z_) × (2π/u_a/z_), the Γ-X path corresponds to the BLG periodicity along the EFW and Γ-Y to the SL periodicity. Point D in the case of ZSL marks the *K*-valley of BLG along the zigzag direction.

**Figure 3:**
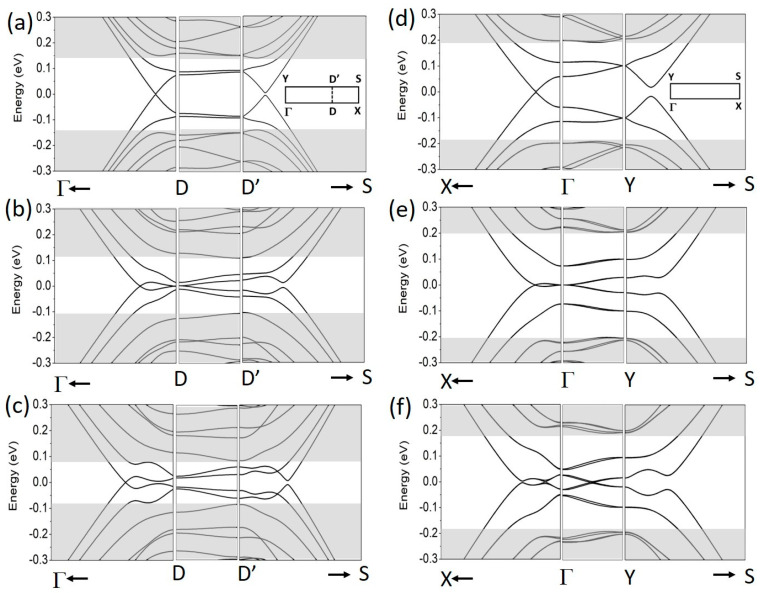
The energy structure in the BZ of the 1D SL as defined in Figure 1a,b. (**a**–**c**) ZSL case, *d* = 25. (**d**–**f**) ASL case, *d* = 30. The gate regimes: (**a**,**d**) R1, (**b**,**e**) R2, (**c**,**f**) R3. *V* = 0.15 eV and Δ = 0.05 eV. In cases (**a**–**c**), the Γ-X path corresponds to the zigzag direction and D indicates the Dirac cone at 2/3 Γ-X. In cases (**d**–**f**), Γ-X corresponds to the armchair direction and the Dirac cone appears at Γ. Shaded areas mark the effective band continua. The insets in (**a**,**d**) show schemes of *k*-paths in the BZs.

The band structures for three different gate regimes are presented in Figure 3. For better visualization of the minibands, the size of mBZ in the direction corresponding to the SL periodicity is artificially enlarged. Panels (a–c) correspond to the ZSL case. In the gate regime R1, the minibands (in the D-D’ path of the BZ) are flat and away from the *E_F_*. In gate regime R2, the minibands touch at the *E_F_* in the D point, and surprisingly, they separate again when the third gate regime R3 is introduced and the effective energy gap diminishes. The situation is very much different in the ASL case (panels (d–f)). The minibands (in the Γ-Y path) are doubly degenerate, less flat, and cross in the gate regime R3. In the first gate regime, the one-dimensional currents can flow only along the EFWs, but in the third regime, the minibands that cross at the *E*_F_ are also conductive. Note also that the minibands in the D-D’ and Γ-Y paths span in the same energy range as the resonance bands of the single EFW case, shown in Figure 2.

**Figure 4:**
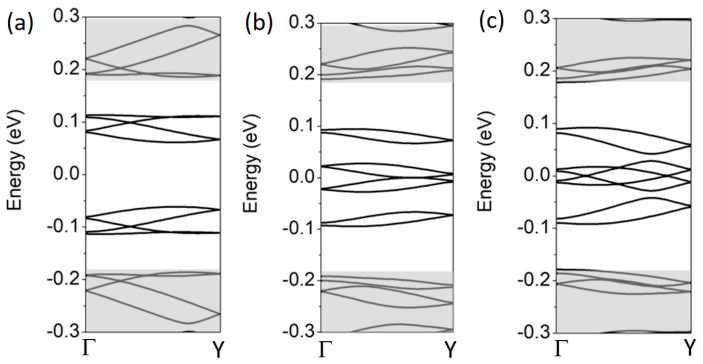
The band structure along the Γ-Y line of the 1D ASL, as in Figure 3d–f, but for *d* = 31. The gate regimes: (**a**) R1, (**b**) R2, (**c**) R3.

A comment is required about the band degeneracy in the ASL case. As mentioned above, the topological bands along the armchair direction are doubly degenerate. This degeneracy need not be sustained if we go along the zigzag direction. The lifting of the degeneracy of minibands in the Γ-Y path is easily visible in Figure 4, where the results for *d*_a_ = 31 are shown. The degeneracy (or quasi degeneracy) that occurs in the *d*_a_ = 30 case comes from the folding properties of the graphene spectrum for lengths that are three times the simple unit in the zigzag direction [40,41].

### 3.3. Two-Dimensional Superlattices

**Figure 5:**
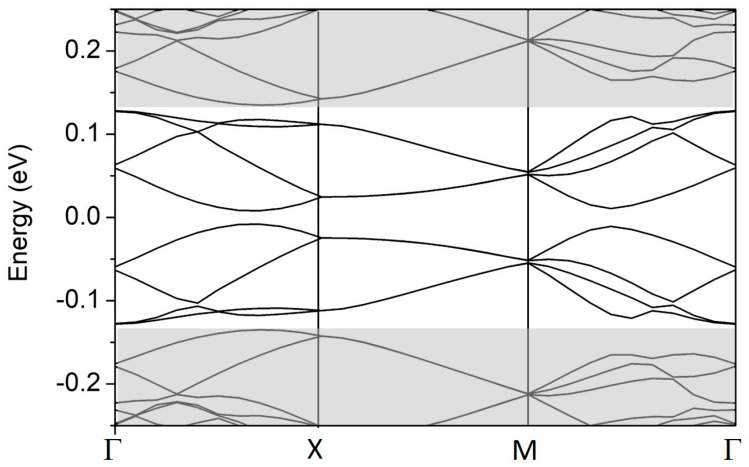
The band structure in the mBZ of the 2D BLG A/B SL, as defined in Figure 1b, in the R1 gate regime. EFWs along the zigzag and armchair directions, *d* = 17 and *V* = 0.15 eV.

In the case of 2D SL, the unit cell is built of four A/B blocks, as schematically shown in Figure 1c. In the calculations, each block has the size *d*_a/z_ = 17. The resulting 2D SL unit cell size is 150 Å × 87 Å and contains 9248 carbon atoms. The minibands calculated for the first gate regime in one-quarter of the mBZ are shown in Figure 5. The gray areas mark minibands originating from the conduction- and valence-band continua. The Γ-X and X-M paths correspond to the zigzag and armchair directions, respectively. The minibands that fall into the effective energy gap come from the topological bands. As expected, there are eight such minibands in the Γ-X and M-Γ paths. All of them are doubly degenerate for the reasons given above, where the energy structure of a pair of EFW in the armchair direction was discussed. However, these bands are four-fold degenerate in the X-M path. The additional degeneracy is due to the mirror symmetry relative to the zigzag direction: the reflection changes the stacking order and, at the same time, reverses the gate polarization. The minibands do not cross at the *E_F_*, so there is no metallic phase.

**Figure 6:**
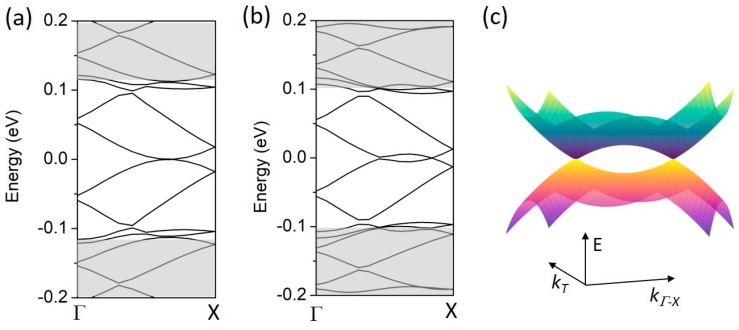
The band structure along the Γ-X line for the 2D BLG A/B SL as in Figure 5 but for R2 (**a**) and R3 (**b**) gate regimes. *V* = 0.15 eV and Δ = 0.05 eV. (**c**) An illustrative 2D visualization of the band crossing points at *E_F_* in (**b**). The *k_Γ-X_* and *k_T_* directions are perpendicular; E is the energy axis.

The situation changes when gate regimes R2 and R3 are applied. In the second regime, the minibands touch at *E_F_* in the Γ-X path, as shown in Figure 6a. However, the band crossing, shown in Figure 6b, is the most significant result for the third gate regime. The band crossing occurs only at the Γ-X path; a small energy gap is still maintained on the other paths, i.e., X-M and M-Γ, as in the case of the R1 regime (see Figure 5). This effect implies the presence of a metallic phase in two dimensions, originating from topological gapless states of the gated bilayer. It has been observed that, close to the crossing points, the minibands extend in a slightly parabolic way (almost linearly) in the *k_T_* direction parallel to the X-M line, which is perpendicular to the Γ-X direction. In particular, the band above the *E_F_* bends up for ±*k_T_* (where *k_T_* = 0 lies at the Γ-X line) and the band below the *E_F_* bends down. This is illustrated in Figure 6c, where the *k_T_* and the Γ-X directions are explicitly shown. Therefore, the two-dimensional minibands cross at four points of the mBZ, and the crossings resemble Dirac cones of graphene.

**Figure 7:**
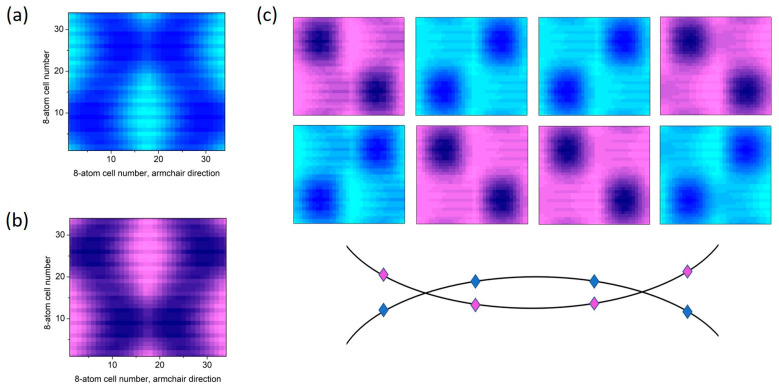
Charge-density distributions (in the 2D SL square unit cell) of the two minibands closest to the *E_F_*. (**a**,**b**) Gate regime R1 (see Figure 5), *k* in the middle of the Γ-X path; blue and pink colors correspond to the bands above and below the *E_F_*, respectively. (**c**) Gate regime R3 (see Figure 6b) at four *k* values for the two crossing bands at the Γ-X path. Different distributions in (**c**) correspond to band wave functions marked as color diamonds in the bottom panel, illustrating the crossing bands. The unit cell, which is schematically shown in Figure 1c, is 34 × 34 u_a/z_. Each pixel summarizes the charge density in the eight-atom unit cell. The EFWs are located both at the edges of the 2D cell and at the horizontal and vertical intersections through the center.

In the case of single-stacking or electric-field walls, the charge densities of gapless states are localized mainly at the interfaces, even if the walls are disturbed [4,6,42,43,44]. We have checked that this is also the case for the topological minibands of 1D SL in the R1 gate regime. When the gate regime changes to R3, the localization also changes and becomes more bulk-like. It happens because the energies of minibands fall into the range of conduction- and valence-band continua. In the case of 2D SL, the bulk-like character of the topological minibands (the ones shown in Figure 5) already dominates in the R1 gates regime, as shown in Figure 7a,b. The effect is especially pronounced in the R3 regime. This is visualized in Figure 7c, where the charge-density distribution in the 2D SL unit cell is shown for several *k* points for the bands crossing at *E_F_*.

## 4. Summary and Conclusions

Using atomistic, tight-binding approximation, we have studied the electronic band structure of 1D and 2D superlattices defined in the bilayer graphene by a spatially modulated perpendicular electric field. The electric field translates into different gate potential values applied to the BLG layers and leads to the opening of the energy gap. The change in the electric field, particularly its polarization in different regions of BLGs, creates domain walls with one-dimensional conducting chiral states localized therein. We have considered electric-field walls arranged along the armchair or/and zigzag directions. Three different field regimes have been investigated: R1 with gate polarization reversed across the walls and an absolute energy gap present in the entire system, R2 with the conduction-band edge in one region merging with the valence-band edge of the neighbor region, and R3 with overlapping valence and conduction bands from the neighbor regions.

In the first regime and the 1D SL, the chiral bands transform into flat minibands corresponding to the SL periodicity, but the metallicity is still governed by the one-dimensional gapless states along the domain walls. In the 2D SL, the chiral states decompose into a series of minibands that fall into the energy gap and the system is no longer metallic. The minibands can merge or even cross at the EF in the second and third gate regimes. The crossing points resemble the Dirac cones of graphene. The crossing minibands provide metallicity in two dimensions, which now is of topological origin. One can also think about possible applications: by manipulating the electric field in some regions of 1D and 2D EFW superlattices, one could open or close conductivity in different directions of the BLG.

## Data Availability

The original contributions presented in the study are included in the article, further inquiries can be directed to the corresponding author.
